# Human antibody response to dengue virus: implications for dengue vaccine design

**DOI:** 10.1186/s41182-016-0004-y

**Published:** 2016-03-14

**Authors:** Meng Ling Moi, Tomohiko Takasaki, Ichiro Kurane

**Affiliations:** Institute of Tropical Medicine, Nagasaki University, Nagasaki, Japan; National Institute of Infectious Diseases, Tokyo, Japan

**Keywords:** Dengue virus, Disease protection, FcgammaR-expressing cells, Antibody-dependent enhancement, Vaccine

## Abstract

Dengue, a global health threat, is a leading cause of morbidity and mortality in most tropical and subtropical countries. Dengue can range from asymptomatic, relatively mild dengue fever to severe and life-threatening dengue hemorrhagic fever. Disease severity and outcome is largely associated with the host immune response. Several candidate vaccines in clinical trials appear promising as effective measures for dengue disease control. Vaccine development has been hampered by safety and efficacy issues, driven by a lack of understanding of the host immune response. This review focuses on recent research findings on the dengue host immune response, particularly in humans, and the relevance of these findings to challenges in vaccine development.

## Background

Dengue virus (DENV) annually infects up to 390 million people living in tropical and subtropical areas [1]. It is estimated that up to half of the world’s population is at risk for DENV infection [2]. In recent years, several countries with subtropical regions, including France, Croatia, and Taiwan, reported autochthonous transmission of DENV [3–5]. DENV cases in Japan have been on the rise; 9 imported cases were reported in 1999 but increased to 249 cases in 2013. In 2014, the first autochthonous DENV transmission in 70 years was reported in Japan, where a total of 162 local DENV patients were reported. In addition, there were 179 imported cases in 2014, bringing the total DENV cases in 2014 to 341, the highest number of cases in recent years [6, 7]. Factors contributing to the rapidly increasing DENV cases over the past 70 years include expanding habitats of the mosquito vectors, *Aedes aegypti* and *Aedes albopictus*, due to urbanization and climate change, and increase in population. *A. aegypti*, a mosquito prevalent in the tropics, is closely associated with large dengue epidemics due to its ability to efficiently transmit DENV. In contrast, the habitat range of *A. albopictus* is not limited to the tropics but includes the temperate subtropical regions of Europe, North America, and East Asia. Because of the widespread distribution of *A. albopictus*, there is a potential threat of dengue spreading to wider geographical areas.

DENV belongs to the genus *Flaviviridae*, a group comprised of antigenically closely related viruses that cause disease in humans, including the Japanese encephalitis virus (JEV), yellow fever virus (YFV), and West Nile virus (WNV). The genome of DENV is a single, plus-stranded RNA which encodes three structural proteins (capsid [C], envelope [E], and preM proteins) and seven nonstructural proteins (NS1, NS2A, NS2B, NS3, NS4A, NS4B, and NS5). Infection with any of the four DENV serotypes that are antigenically and genetically related causes symptoms ranging from acute febrile illness to severe manifestations that include bleeding and organ failure. Without appropriate treatment, the mortality rate of dengue hemorrhagic fever (DHF) is approximately 25 %. Currently, there is no specific treatment or vaccine for dengue.

## Clinical signs of dengue

DENV causes a wide range of clinical symptoms, ranging from asymptomatic infection and dengue fever (DF) to the severe forms of illness, DHF, and dengue shock syndrome (DSS). Symptoms typically appear 4–7 days after an incubation period following a bite from an infected mosquito. DF is a self-limited febrile illness characterized by a sudden onset of fever accompanied by a generalized morbilliform rash, headache, myalgias, and retro-orbital pain during the acute phase. The febrile phase generally lasts for a week. Symptoms are also accompanied by thrombocytopenia and leukopenia. A small proportion of dengue patients develop severe dengue (DHF or DSS). In severe dengue, life-threatening symptoms of plasma leakage, severe thrombocytopenia, hemorrhagic manifestations, and coagulation disorders may lead to shock and multiple organ failure during the critical phase of the disease. After the critical phase, most patients recover without any sequelae, although some develop fatigue and depression that lasts for several weeks after the acute phase. In severe dengue, an estimated 500,000 require hospitalization, and the mortality rate is 2.5 %, particularly in infants and young children [2]. Atypical clinical signs and symptoms of dengue include encephalitis, myocarditis, and respiratory distress [8].

## Host immune responses to DENV

Humoral and cellular immune responses may play important roles in both disease pathogenesis and protection [9–12]. The paradoxical role of the host immune response in disease outcome is associated with consecutive infection by two different DENV serotypes. There are four serotypes of DENV, characterized by their antigenicity. The basis for host immunity in the disease outcome involves (1) virus-neutralizing antibodies directed against viral proteins and (2) immune cascades triggered by viral epitope-activated memory T cells [9–12].

Epidemiological studies have found that primary dengue patients, after a short cross-protective period, are at higher risk of developing severe dengue during a consecutive infection with another DENV serotype [12, 13]. The increased risk of developing severe dengue is hypothesized to be associated with non-protective serotype cross-reactive immunity induced after primary DENV infection. The basis for this is that after a primary DENV infection, serotype cross-reactive protective immunity wanes over a few months [14]. Neutralization activity in vitro was absent in diluted patient serum samples, indicating that a certain antibody threshold is needed for virus neutralization [14, 15]. Although serotype cross-reactive neutralizing antibodies confer protection at a certain threshold, waning immunity or a decline in antibody concentration induces susceptibility to infection with another DENV serotype.

Upon secondary infection with a heterogeneic serotype during this period of waning immunity, homotypic serotype-specific memory T and B cells are activated. This early serotype-specific immunity is directed against infection by the prior DENV serotype, but not to the current infecting serotype [9, 10]. During secondary infection, T cell responses directed against the previous infecting serotype do not confer protection and are associated with the triggering of immune cascades that induce severe symptoms [9].

As the disease progresses to the convalescent phase, serotype cross-reactive immunity with high avidity to all four DENV serotypes develops. These broadly reactive antibodies confer protection to all four DENV serotypes. A third or fourth infection may occur in hyperendemic areas, although a majority of these infections are hypothesized to be asymptomatic or mild [11].

### Role of antibodies in pathogenesis of severe dengue

Epidemiological evidence has demonstrated that two groups of individuals are at higher risk of developing severe dengue: (1) adults with waning homotypic immunity and (2) infants born to dengue-immune mothers [13–15]. Serum samples obtained from these individuals during the period of waning immunity were found to enhance DENV infection in vitro [16]. Antibodies in these serum samples possess the ability to enhance DENV infection of Fcgamma (FcγR)-bearing cells in a phenomenon known as antibody-dependent enhancement (ADE) [16–20]. In ADE, subneutralizing levels of antibodies form a virus immune complex that enhances the infection of DENV target cells, the FcγR-bearing hematopoietic cell lines, leading to high levels of viremia [21–23]. Viremia determined by FcγR-bearing cells is up to 10 times greater than that determined by non-FcγR-bearing cells [13]. The resulting high level of viremia triggers the immune system, which in turn is associated with increased disease severity [22, 23].

Serum samples from convalescent DENV patients typically demonstrate ADE activity at higher dilutions and neutralizing activity at lower dilutions, indicating that antibodies induced after infection possess two contrasting activities [17]. In patients with acute secondary infection, undiluted serum samples demonstrated ADE activity against the infecting serotype, suggesting that ADE occurs in vivo [14–16]. ADE has also been demonstrated in animal models of secondary infection, in animals passively transferred with infection-enhancement antibodies, and in a maternally acquired heterotypic dengue antibody animal model [24–27].

### Antibodies in protection against DENV infection

Neutralizing antibodies are considered central in protection against DENV infection. Antibodies targeting DENV possess two competing activities related to pathogenicity: virus neutralization and infection enhancement. At higher avidity, antibodies neutralize DENV, whereas lower levels of antibodies enhance DENV infection and hamper virus neutralization [17]. The concept was confirmed in studies of serum samples from dengue patients, in which neutralizing activity was present to several DENV serotypes using non-FcγR-expressing cells, but ADE activity that lowers the overall neutralizing activity was present to the current infecting serotype (DENV-1 and DENV-3), as demonstrated by FcγR-expressing cells (Table 1) [17]. After the cross-protection phase in patients with primary infection, antibodies demonstrate high levels of neutralizing activity to a homotypic serotype, but neutralizing activity against other serotypes is absent. Antibodies with high neutralizing activity to the prior infecting serotype also demonstrate cross-reactivity to other serotypes as well as infection-enhancement activity [7].

**Table 1:** Absence of neutralizing antibody to infecting serotypes as determined by FcγR-expressing BHK cells

Infecting serotype (*n*)^a^	Number of Patients	DENV neutralizing antibody titer (PRNT_50_)							
		BHK cells				FcγR-expressing BHK cells			
		DENV-1	DENV-2	DENV-3	DENV-4	DENV-1	DENV-2	DENV-3	DENV-4
DENV-1 (7)	3^b^	10–80	10–1280	<10–20	<10–20	<10	<10–160	<10	<10
	4	<10	10–320	<10	<10	<10	<10–40	<10	<10
DENV-3 (10)	8^b^	10–80	<10–1280	10–40	<10–40	<10–20	<10–80	<10	<10
	2	<10	80–160	<10	<10	<10	20–40	<10	<10

Data source [17]

^a^Indicates the total number of patients

^b^Indicates the number of patients with neutralizing antibody to infecting serotype as determined by non FcγR-expressing cells

After acute infection, a high proportion of the antibodies is directed against the E protein, and some target the prM and NS1 proteins. The virion envelope is a lipid bilayer, which consists of the E and M proteins. A majority of the induced antibodies target the E protein; therefore, virus neutralization may involve neutralization of the E protein epitopes. The antibodies induced consistently react with the E protein, but there is variability in the reactivity to domains I, II, and III; the considerable variability in reactivity in each of the domains isolated from individual patients indicates that virus neutralization may involve several epitopes [28, 29].

In DENV hyperendemic regions, co-circulation of several serotypes demonstrates an epidemiological pattern in which outbreaks arise from a dominant serotype, in a 2–4-year cycle [30, 31]. These cycles persist because infection with one serotype confers only partial protection and may be driven by low immunity to each of the dengue serotypes, which then allows the virus to persist in the region. In hyperendemic regions, most adults are exposed to two or more serotypes and demonstrate seroconversion.

### Determination of neutralizing activity of an antibody to DENV

As neutralizing activity of an antibody is considered central in providing protection against dengue, levels of neutralizing antibody have been used as a proxy for disease protection. Determination of levels of neutralizing antibody is also important to analyze the immune response after vaccination. Neutralizing antibody to dengue is determined by using the plaque reduction neutralization test (PRNT). The PRNT is regarded as the gold standard for determination of neutralizing antibodies [32]. High titers of neutralizing antibody titers are associated with protection against subsequent DENV infection. However, infection-enhancement activity has been demonstrated to lower neutralizing activity of antibodies in the presence of FcγR [17, 33]. Thus, the use of non-FcγR-bearing cells for assays poses a challenge in defining the true serological correlate to DENV protection in vivo and in vaccine efficacy trials. Because FcγR-bearing monocyte-lineage cells are the major targets of DENV infection in vivo, these cells may better reflect the biological properties of neutralizing antibodies [34]. Primary and established monocyte-lineage cell lines have also been used in neutralization tests [14, 15, 35–38].

Although assays to determine neutralization antibody titers have been developed, the correlate of protection for dengue has yet to be defined. For JEV, a titer of ≥1:10 at PRNT50 has been suggested as a threshold that demonstrates protection [39, 40]. For DENV, titers of ≥1:10 have been demonstrated in convalescent serum samples, but the cutoff has not been determined. Determination of cutoff titer that demonstrates protection is needed to better predict vaccine efficacy.

Although the PRNT is the most reliable test to determine neutralizing antibodies, it is laborious and requires skilled personnel. Considering the magnitude of the dengue problem, standardized high-throughput assays are needed for both research and industrial settings. Early studies using flow cytometry, peroxidase-antiperoxidase (PAP) and an enzyme-linked immunosorbent assay (ELISA) that employs FcγR-expressing cells have proved useful in the determination of neutralizing activity of DENV antibodies [35–38]. Tools that use the viral protein subunits and E protein epitopes are promising, as these tests are rapid compared to the PRNT [41, 42]. However, further studies are needed to validate these tests and the results compared to those of the conventional PRNT, to establish a robust immune correlate for disease protection [32].

## Implications of current findings of the host antibody response for DENV vaccine design

Currently, there are no licensed dengue vaccine which are used clinically. Dengue vaccine research has been carried out for the past 70 years, beginning with the first isolation of the virus during epidemics in Hawaii and Japan. Within a few decades, live-attenuated virus, inactivated vaccine, chimeric flavivirus, DNA, and subunit vaccines have been developed (Table 2). However, vaccine immunogenicity, safety, and efficacy have been recurring issues in their use for control of the disease. Additional challenges hampering clinical development include the lack of a robust immune correlate for dengue, possible inhibitory or enhancing effects that are induced upon natural flavivirus infection and vaccination, and the need for definition of vaccine trial endpoints.

**Table 2:** Characteristics of candidate tetravalent dengue vaccines in various phases of clinical trials

Vaccine type	Manufacturer	Candidate vaccine	Candidate vaccine description	Clinical trial phase	Protective efficacy of vaccine
Attenuated chimera vaccine	(1) Sanofi-Pasteur	CYD-TVD	Recombinant DENV vaccine with yellow fever 17D vaccine strain as backbone and substitution of preM and E protein genes with each of the four DENV serotype	PIII	56.5–60.8 %
	(2) US-CDC/Inviragen/Takeda Pharmaceuticals	DENVax	Recombinant DENV vaccine with DENV-2 as backbone and substitution of preM and E protein genes of DENV-1, DENV-3, and DENV-4	PII	–^1^
	(3) NIAID	TetraVax-DV	Attenuated tetravalent formulation with a deletion of 30 nucleotides from 3′ UTR of DENV-1, DENV-3 (or chimeric DENV-3/DENV-4), DENV-4, and a chimeric DENV-2/DENV-4	PII	–
Attenuated vaccine	Mahidol University/Kaketsuken	–	Attenuated vaccine strains by serial passages in PDK cells	PII	–
Inactivated vaccine	WRAIR, GSK	TDENV-PIV	Whole purified inactivated vaccine	PI	–
Subunit vaccine	Merck (Hawaiian Biotech)	DENV-V180	Truncated DENV E protein	PI	–
DNA vaccine	U.S. Army Medical Research and Material Command/WRAIR	TVDV	Plasmids encoding the prM/E genes of each DENV serotype	PI	–

^a^Indicates no data

The observation of the development of severe dengue during heterotypic secondary infection implies the need for a vaccine that possesses the ability to simultaneously induce long-term protective immunity to all four serotypes. Multiple vaccine formulations are currently under development, and several candidates are currently in preclinical and clinical stages. Currently, the live-attenuated tetravalent chimeric YFV/DENV vaccine (CYD-TDV) is in the most advanced clinical trial stage (phase III) [43]. The phase I and II studies of the CYD-TDV vaccine have demonstrated that it is safe and tolerated, with confirmation of seroconversion and virological efficacy [44]. In multisite phase III clinical trials conducted in Asia and Latin America, the overall efficacy of the CYD-TDV candidate vaccine ranges from 30 to 64 % against DENV infection. The incidence of severe dengue (>80 % efficacy) and hospitalization was also lower in the vaccine cohort study. Surprisingly, vaccine efficacy against DENV-2 in comparison to the other serotypes was consistently low, ranging from 9 to 42 % [43–46]. The overall efficacy also varied with study site, suggesting that efficacy may be associated with epidemiological patterns and the host immunity baseline. While a lower incidence of severe dengue was seen in the vaccine cohort, results from the study demonstrated that seroconversion did not consistently reflect protective immunity [44].

Recent DENV vaccine clinical trials and patient cohort studies have found that despite the presence of seroconversion (PRNT50 >1:20) in vaccines, protection against DENV-2 was minimal [44]. Although conventional PRNT assays used in these studies are considered reliable in determining neutralizing antibodies as a proxy for disease protection, the assay uses FcγR-negative cells. A novel PRNT assay has demonstrated that neutralizing activity of some serum samples from dengue patients was absent or present at much lower titers when determined using FcγR-expressing BHK cells, as compared to non-FcγR-bearing cells [17]. Interestingly, neutralizing activity to the current infecting serotype was not detected by using FcγR-bearing cells but was present when non-FcγR-bearing cells were used for the assay (Table 1). These studies suggest that DENV-2 infection breakthrough despite seroconversion could be caused by the dominance of antibodies that might not confer virus neutralization in the presence of FcγR.

Alternatively, it is hypothesized that neutralizing activity of an antibody displays differential activity against DENV genotypes. In vitro and animal studies have demonstrated that differential neutralizing activity when Asian and American DENV-2 strains are induced after exposure to DENV-1 [47, 48]. Although multiple genotypes exist in a DENV serotype, the differences in the antigenicity of each genotype in relation to the resulting protection against different genotypes need further characterization.

In addition to protective efficacy issues, hypothetical safety concerns include (1) possible enhancement of clinical response to live-attenuated vaccine in individuals with a flavivirus-immune background, (2) non-protective or waning immunity in vaccinated individuals that could potentially increase the risk of severe dengue, and (3) insufficient attenuation of vaccine strains. The CYD-TDV candidate vaccine trial has demonstrated that the vaccine is safe, and efficacy has been demonstrated through lesser number of virologically confirmed cases within the 2-year study period [46]. However, long-term results would provide insights into waning immunity in vaccinated individuals and the risk of severe dengue [45]. Additionally, further studies on the immune response to dengue vaccines in non-endemic areas would provide a better understanding of the host immune response in individuals without a dengue-immune background.

With half of the world population at risk of DENV infection, an effective dengue vaccine that confers long-term protective immunity to all four dengue serotypes would contribute significantly to disease control. Other considerations in dengue disease control would include suitability of the vaccination program, cost constraints, and identification of disease burden. The CYD-TDV (Dengvaxia) vaccine has been approved as the first registered dengue vaccine in Mexico in December 2015. The CYD-TDV vaccine is also currently under review for clinical introduction in other endemic regions and is anticipated to reduce dengue burden. Follow-up observations on the CYD-TDV vaccine efficacy would however be needed, particularly on breakthrough infections and severe dengue in vaccine recipients. Other promising vaccine candidates in clinical and preclinical trials include virus-vectored and VLP-based vaccines. These next-generation vaccine candidates may offer improved approaches to disease control as they have been demonstrated to be safe due to the non-infectious nature of the vaccine, highly immunogenic, and could be modified rapidly to match recent epidemic strains. With dengue disease burden being the highest in developing countries, a cost-effective, safe vaccine that confers protection with a single dose would be ideal in disease control programs.

## Conclusions

Dengue epidemics continue to be a serious public health problem in many areas of the world. Disease control remains a challenge in the absence of a vaccine and specific therapies. Development of tools and robust animal models to advance understanding of the host immune response will facilitate a better understanding of vaccine efficacy and safety issues and contribute to the development of dengue vaccine and treatment programs.
